# Effect of heating and cooling combination therapy on patients with chronic low back pain: study protocol for a randomized controlled trial

**DOI:** 10.1186/s13063-015-0800-4

**Published:** 2015-06-26

**Authors:** Eun-Jung Kim, Young-Doo Choi, Chi-Yeon Lim, Kyung-Ho Kim, Seung-Deok Lee

**Affiliations:** College of Korean Medicine, Dongguk University, Gyeongju, South Korea; Department of Medicine, Dongguk University, Gyeongju, South Korea; Department of Acupuncture and Moxibustion, Dongguk University International Hospital, Siksa-dong, Ilsandong-gu, Goyang, Gyeonggi-do Korea

**Keywords:** Heating and cooling combination therapy, Low back pain, Randomized controlled trial (RCT)

## Abstract

**Background:**

Clinicians often apply heating or cooling stimulation for treatment of musculoskeletal pain. However, scalding, frostbite and skin ulcers may occur from the excessive use of either therapy alone. Heating and cooling combination therapy may be a suitable alternative for treatment of musculoskeletal diseases, although insufficient research has documented the safety and efficacy of such therapy. The purpose of this clinical trial is to determine the efficacy and safety of heating and cooling combination therapy for treatment of chronic low back pain.

**Methods/Design:**

This is a multicenter, parallel-group, double-blinded, randomized controlled trial to evaluate the efficacy and safety of a heating and cooling combination therapeutic device (OCH-S100) in patients with chronic low back pain. Eighty participants with chronic low back pain will be recruited from two hospitals in South Korea (Dongguk University Ilsan Oriental Hospital and Dongguk University Bundang Oriental Hospital). Enrolled patients will be randomly divided into a treatment group and a sham group. Patients in both groups will be given 10 treatments (15 min per treatment) over 4 weeks. The protocol will consist of five cycles of heating/cooling therapy (maximum: 45 °C, minimum: 15 °C) in the treatment group, and five cycles of sham therapy (maximum: 1 °C above skin temperature, minimum: 1 °C below skin temperature) in the sham group. The primary outcome measure is change from baseline in the 100 mm Visual Analogue Scale (VAS) for pain after 4 weeks. There are six secondary outcome measures that consider disability or range of motion (ROM).

**Discussion:**

This research will determine the efficacy and safety of heating and cooling combination therapy on chronic low back pain. The results of this trial may have important implications for the more widespread use of heating and cooling combination therapy for treatment of musculoskeletal pain.

**Trial Registration:**

NCT02289170 (14 October 2014)

## Background

Heating and cooling therapies are widely used to treat musculoskeletal pain [1]. For example, in the United States, 75 % of patients with low back pain are treated with heating therapy and 7 % with cooling therapy [2].

Heating therapy increases tissue temperature, blood flow, metabolism and connective tissue extensibility [1]. There are also reports that heating therapy increases the activity in all of the Ia and many of the Ib afferents, decreases the activity of most group II spindle afferents, decreases muscle spasms, and increases the speed of nerve conduction [3]. Other studies indicated that heating therapy is more effective than placebo in relieving pain, decreasing disability, decreasing muscle tension, and improving range of motion [4, 5].

There is evidence that cooling therapy decreases tissue blood flow due to vasoconstriction, and that it also reduces tissue metabolism, oxygen utilization, and inflammation [1, 6]. Cooling therapy decreases the velocity of nerve conduction in superficial tissues by slowing the firing of muscle spindle afferents and reflex responses, thus decreasing muscle spasms and pain [7]. Use of cooling therapy for musculoskeletal problems can also reduce intake of painkillers because it reduces pain and body fluid penetration [8].

However, the overuse of cooling therapy or heating therapy due to physiological adaptation can lead to skin damage such as scalding, frostbite, and ulcers [1, 6]. We developed a heating and cooling combination therapy device (OCH-S00) that reduces the probability of skin damage. This device maximizes the stimulation and reduces the potential harm from heating and cooling by use of a probe to reduce sensory adaptation.

Thermo-receptors are sensitive to different temperatures. Thus, TRPA1 is mainly responsible for painful cold sensation below 18 °C, TRPM8 for cool sensation at 23 to 28 °C, TRPV2 for warm sensation at 30 to 39 °C, and TRPV1 for painful hot sensation above 43 °C [9]. The heating and cooling device described here stimulates the cold, cool, warm and hot receptors by applying temperatures in the range of 15 to 45 °C, well below the burn point. Thus, this device is expected to have anti-inflammatory effects, increase muscle relaxation, decrease edema and provide pain relief, without damaging the skin.

The purpose of this research is to evaluate the safety and efficacy of the OCH-S100 heating and cooling combination therapy device on patients with chronic low back pain.

## Methods/Design

### Study design

This research of the clinical efficacy and safety of heating and cooling combination therapy is a multicenter, randomized, double-blinded, parallel-group study. All enrolled subjects are patients with chronic low back pain. This research protocol was externally peer reviewed by Korean Ministry of Food and Drug Safety (KMFDS) and Korean Health Industry Development Institute (KHIDI). Two clinical research centers in Korea will participate in this trial: Dongguk University Ilsan Oriental Hospital and Dongguk University Bundang Oriental Hospital. This research protocol was approved by Dongguk University Ilsan Oriental Hospital’s Institutional Review board (IRB) and Dongguk University Bundang Oriental Hospital’s IRB. Each subject will provide informed consent before enrollment.

### Recruitment

Participants will be recruited through advertisements in local newspapers and on bulletin boards at each trial center. The target sample size is 80 subjects. Telephone screening will be performed before the trial to determine if subjects fulfill the selection criteria. Final enrollment will be determined by an orthopedist and a radiologist through physical exams, radiological tests, blood and urine tests, and relevant questionnaires.

### Participants

All outpatients who are 20 to 75 years of age and have suffered from low back pain in the previous 3 months or more and had ratings over 40 mm on a 100 mm Visual Analog Scale (VAS) for pain will be screened. Subjects will be excluded if any of the following are present: (i) trauma or surgery in the lumbar region in the 6 months prior to enrollment; (ii) low back pain accompanied by sciatica; (iii) systemic disease or severe dysfunction due to another medical problem (cancer, infection, *etcetera*); (iv) history of using medication(s), traditional oriental medical care, or physiotherapy for treatment of low back pain in the 4 weeks prior to enrollment; (v) neurological disease, such as topical or whole body hypoesthesia, that may affect temperature sensation (stroke, peripheral neuropathy, *etcetera*); (vi) hypersensitivity to heating and cooling stimulation; (vii) pregnancy or lactating; or (viii) participation in other clinical trials in the 3 months prior to or after enrolling in this trial.

### Randomization and blinding

Randomization will be performed by an independent statistician using a computerized random-number generator through the block-randomization method of SAS version 9.1 (SAS Institute Inc. Cary, NC, USA). Separate randomization files will be created for each study site. Allocation concealment will be maintained by use of opaque sealed envelopes. After the researcher screens a patient for inclusion and receives signed informed consent, the next envelope in the sequence will be accessed and opened by the certified Korean Medicine Doctor who supervises the medical device, immediately prior to treatment. A total of 80 participants will be randomly and blindly assigned to a treatment group or a sham group. All subjects, practitioners, and the outcome assessor will be blinded to group assignment. Blinding of the certified Korean Medicine Doctor who supervises the medical device and sets up the treatment (medical equipment manager) is not possible. The patients will be told: “You will be treated with one of two heating and cooling combination treatments. One has a large range of temperature change and the other has a small range of temperature change.”

### Treatment and assessment

Subjects in both groups will be treated 10 times over 4 weeks. The treatment term of 4 weeks was based on previous studies of the treatment of general chronic low back pain [10]. Each treatment will be 15 min and there will be two to three treatments per week, based on the protocol of previous research on heating and cooling combination treatment [11]. The outcome assessor (who does not participate in the treatment) will measure the degree of pain sensitivity by pressing eight acupoints (four on each side of the waist; BL23, BL24, BL25, and BL26) that are frequently used to assess low back pain [12–16]. This assessment will employ a pressure algometer, with a circular and flat probe of 1 cm diameter (Commander Algometer, JTECH Medical, Midvale, Utah, USA), to identify the two most painful acupoints. Before treatment, the medical equipment manager will measure the skin temperature of each patient in the treatment and sham groups using thermometer (Superfast Thermapen Pro-surface, ETI, England) (Table 1).

**Table 1:** Content of outcome measurement

	Treatment Number											
Outcome measure	B	1	2	3	4	5	6	7	8	9	10	F
Primary outcome												
VAS	X	X	X	X	X	X	X	X	X	X	X	X
Secondary outcomes												
KODI		X					X				X	X
KRMDQ		X					X				X	X
TMBQ		X					X				X	X
Modified Schöber test		X					X				X	X
Finger-to-floor distance		X					X				X	X
Finger-to thigh distance		X					X				X	X

*B* baseline, *F* follow-up, *VAS* Visual Analog Scale, *KODI* Korean Oswestry disability index, *KRMDQ* Korean Roland Morris Disability Questionnaire, *TMBQ* Traditional Medicine Back pain Questionnaire

#### Heating and cooling combination therapy

The patients in this group will receive heating and cooling combination therapy. The probe is attached to the acupuncture points for 15 min, and its temperature will be cycled five times, from the maximum of 45 °C to the minimum of 15 °C. This temperature range was chosen to provide stimulation of cold, cool, warm and hot receptors.

#### Sham therapy

The patients in this group will receive sham heating and cooling combination therapy under identical conditions, except for probe temperature. The probe’s temperature will be cycled five times, from 1 °C above skin temperature to 1 °C below skin temperature.

During the 4-week study, patients will be prohibited from using any treatment that could influence the results, such as analgesics, nonsteroidal anti-inflammatory drugs, muscle relaxants, oral corticosteroids, antidepressants, steroid injections, high frequency heat coagulation, physiotherapy, acupuncture, moxibustion and surgery. Patients who use any of these treatments will be excluded. All patients will be questioned about medication usage during each treatment session.

### Quality assurance

We will educate all practitioners and set qualification standards to make sure the patients are treated with high standards and in accordance with the trial protocol.

Treatment will be administered by doctors of Korean Medicine who have extensive clinical experience with heating and cooling stimulation therapy and moxibustion and the following qualifications: (i) graduate of a 6-year full-time course in Korean Medicine, taught as a college program; (ii) certified by the Korean Ministry of Health and Welfare as a Korean Medicine Doctor; (iii) more than 1 year of postgraduate clinical training in an Oriental medicine hospital; and (iv) completion of the first-year residency program in our Department of Acupuncture and Moxibustion.

Practitioners and medical equipment managers will attend a 2-day training workshop about the treatment programs and will be educated on selection of acupoints by using a pressure algometer. Medical equipment managers will also be educated on measuring skin temperature and setting the treatment and sham devices. A written protocol and standardized recording documents will be provided. Because the relationship between a practitioner and patient could influence the results, all such interactions will be strictly limited, except when reporting side effects. All practitioners and outcome assessors will be blinded to group allocations, and medical equipment managers will not participate in the interventions. To assure blinding of patients, we will put a small fabric curtain next to the device to block the patient’s view.

### Analysis of data

A VAS will be used to assess the effect of treatment. The primary endpoint is the change in the 100 mm Pain VAS from baseline (before treatment) to 4 weeks [10, 12, 17]. The 100 mm VAS is widely used because it provides reliable and valid measurements of pain intensity [18]. Each patient will be asked to check their pain intensity along an ungraded 100 mm horizontal line, where ‘0’ represents ‘no pain’ and ‘100’ represents ‘unbearable pain’.

The secondary endpoints are change from baseline in three evaluations of functional disability (Korean Oswestry disability index [KODI], Korean Roland Morris Disability Questionnaire [KRMDQ], and Traditional Medicine Back pain Questionnaire [TMBQ]) and three evaluations of range of motion (ROM) (modified Schöber test, finger-to-floor distance, and finger-to-thigh distance). These parameters will be measured at baseline, 2 weeks, 4 weeks, and at 4 weeks after the last treatment.

The KODI has 10 criteria related to daily living activities and each item has six possible responses, ranging from ‘no problem’ to ‘not possible’. The KODI focuses on a limitation in the range of physical functions, including standing, walking, lifting, sitting, lying down, dressing, and personal care [18]. We excluded questions about sexual activities because many subjects do not provide honest answers about their sex lives, and it is hard to account for subjects who are single. The KRMDQ contains 24 yes/no queries and also focuses on limitations in physical functions, including walking, bending over, sitting, lying down, dressing, sleeping, self-care, and daily activities. The KRMDQ is easy to understand, interpret and score. Higher scores in the KRMDQ and KODI indicate greater disability [18]. We will use Korean versions of these surveys, which have documented reliability and validity [19, 20]. The TMBQ evaluates low back pain and is based on traditional Korean medicine [21].

The modified Schöber test measures the curvature of the spine. In this test, the lumbosacral junction is marked on the back; a second mark is placed 5 cm below the junction, and a third mark is placed 10 cm above the junction. Then the subject is asked to bend forward as far as possible, and the new distance between the second and third marks is measured [22]. We will also use a digital flexibility testing device (Standing Trunk Flexion meter TKK5403, Takei Co., Japan) to assess the trunk flexibility, finger-to-floor distance (distance between the fingertips and the floor after forward flexion with knees straight, while on a 20-cm high box) [23], and finger-to-thigh distance (distance between the fingertips and the floor after lateral bending with knees straight, while on a 20-cm high box) [24].

### Safety assessments

Any physical or clinical changes will be reported by the assessor, practitioner, and patient at each visit. All symptoms, date of onset, and duration will be recorded.

### Sample size

The primary endpoint will be change in 100 mm VAS score after 4 weeks of treatment. Liu *et al*. [25] previously showed that the 4-week change was 7.6 for the test device and 3.9 for the control device. Patients will be randomized to the test and sham groups in a 1:1 ratio. Assuming a reference standard deviation (SD) of 5.37, power analysis indicates that a sample size of 34 has an 80 % probability of yielding a significant difference at the level of 0.05. In order to achieve stronger results, a one-sided 97.5 % lower confidence limit for the treatment difference (VAS on test device - VAS on sham device) will be greater than 0. We will allow for a 15 % dropout rate by randomization of 40 subjects to each group.

### Statistical analysis

All data will be analyzed using SAS version 9.1 (SAS institute Inc. Cary, NC, USA). All statistical tests will be two-sided, and the level of significance will be set at 0.05. Continuous variables will be expressed as means ± SDs and categorical variables as numbers and percentages. For analysis of the primary endpoint, the two-sided 95 % confidence interval will calculated by analysis of covariance (ANCOVA) and the model will include fixed-effect for study site. Student’s *t*-test will be used for evaluation of primary and secondary end-points. Categorical data will be analyzed using Pearson’s chi-square test or Fisher’s exact test, as appropriate.

For safety analysis, the numbers and percentages of patients will be presented in a frequency table. Patients with adverse events will be listed along with their medical histories and concomitant medications, with start and stop dates. Categorical variables will be summarized by frequency tables, and continuous laboratory variables, and changes from baseline will be expressed by summary statistics. In addition, the number and percentage of patients with each value below, within and above the normal range will be tabulated, and “shift tables” (summaries of individual changes in laboratory parameters) will be generated.

## Discussion

This clinical research aims to evaluate the safety and efficacy of OCH-S100, a device designed to administer heating and cooling combination therapy and provide relief for patients with chronic low back pain.

Heating and cooling therapy is widely used for treatment of musculoskeletal pain in clinical practice [7, 11]. The beneficial effects of this therapy may be mediated by changing blood flow, decreasing inflammation, induction of contraction and relaxation of blood vessels, decreasing edema, or decreasing stiffness of the muscle [11], but the underlying mechanism has not yet been proven. Moreover, there is only limited research on the efficacy of heating and cooling combination therapy. Thus, the clinical research described here is one of the few studies to test rigorously the efficacy and safety of heating and cooling combination therapy. Our research was designed to be rigorous, in that it will employ two study centers, double blinding of all assessors and practitioners, prior education of all practitioners, and fabric curtains to block patients’ view of the device. Moreover, a certified Korean Medicine Doctor with extensive experience will be treating all patients.

The device used in this research can precisely deliver temperatures from 15 °C to 45 °C, temperatures that do not burn or damage the skin. Therefore, this research will be able to document the effect of stimulation of the cold, cool, warm, and hot receptors on patients with chronic low back pain.

## Trial status

Patient recruitment is ongoing. Enrollment and trial completion is expected by the end of 2015.

## Abbreviations

ANCOVA: analysis of covariance
IRB: Institutional Review Board
KODI: Korean Oswestry Disability Index
KRMDQ: Korean Roland Morris Disability Questionnaire
ROM: range of motion
SD: standard deviation
TMBQ: Traditional Medicine Back pain Questionnaire
VAS: visual analog scale
